# Genetic Association Study between*STK39* and *CCDC62/HIP1R* and Parkinson’s Disease

**DOI:** 10.1371/journal.pone.0079211

**Published:** 2013-11-27

**Authors:** Nan-Nan Li, Eng-King Tan, Xue-Li Chang, Xue-Ye Mao, Jin-Hong Zhang, Dong-Mei Zhao, Qiao Liao, Wen-Juan Yu, Rong Peng

**Affiliations:** 1 Department of Neurology, West China Hospital, Sichuan University, Chengdu, Sichuan, China; 2 Department of Neurology, Singapore General Hospital, National Neuroscience Institute, Singapore, Singapore; 3 Duke–NUS Graduate Medical School, Singapore, Singapore; 4 Department of Internal Medicine, Wangjiang Hospital, Sichuan University, Chengdu, Sichuan, China; Oslo University Hospital, Norway

## Abstract

**Background:**

The first large-scale meta-analysis of published genome-wide association studies (GWAS) in Parkinson’s disease (PD) identified 5 new genetic loci (*ACMSD*, *STK39*, *MCCC1/LAMP3*, *SYT11*, and *CCDC62/HIP1R*). Very recently, a large-scale replication and heterogeneity study also reported that *STK39* and *CCDC62/HIP1R* increased risk of PD in Asian and Caucasian populations. However, their roles still remain unclear in a Han Chinese population from mainland China.

**Methods:**

We examined genetic associations of *STK39* rs2102808 and *CCDC62/HIP1R* rs12817488 with PD susceptibility in a Han Chinese population of 783 PD patients and 725 controls. We also performed further stratified analyses by the age of onset and accomplished in-depth clinical characteristics analyses between the different genotypes for each locus.

**Results:**

No significant differences were observed in the minor allele frequency (MAF) among cases and controls at the two loci (*STK39* rs2102808: OR = 1.06, 95% CI = 0.91, 1.23, *P* = 0.467; *CCDC62/HIP1R* rs12817488: OR = 0.88, 95% CI = 0.76, 1.01, *P* = 0.072). Subgroup analyses by the age of onset also showed no significant differences among different subgroups of the two loci. In addition, minor allele carriers cannot be distinguished from non-carriers based on their clinical features at the two loci.

**Conclusions:**

We are unable to demonstrate the association between *STK39* and *CCDC62/HIP1R* and PD susceptibility in a Han Chinese population from mainland China. Additional replication studies in other populations and functional studies are warranted to better validate the role of the two new loci in PD risk.

## Introduction

Parkinson’s disease (PD, OMIM #168600) is the second most common adult-onset neurodegenerative disorder involving not only motor impairment but also deficits in behavior, cognition, and daily function [1]. Fewer than 5% of all PD cases can be attributed to genetic mutations in α-synuclein and the other known PD genes [2]. However, the vast majority of PD cases are considered idiopathic, whose specific pathogenesis remains to be elucidated. Understanding the genetic architecture of PD might provide valuable insights into individual risk predictions and gene therapy for PD in the near future.

With the recent developments of high throughput genotyping and genome-wide association studies (GWAS), tremendous progress was made in our understanding of the genetic basis for this complex disorder [3]. Up to now, several GWAS and many candidate gene studies have provided consistent associations with *SNCA* and *MAPT* [4]–[16], with some evidence for the role of *BST1*, *GAK/DGKQ*, and the *HLA* region in PD susceptibility [7], [11]–[13], [16]. The three recent meta-analyses reported eleven new loci: *ACMSD*, *STK39*, *MCCC1/LAMP3*, *SYT11*, *CCDC62/HIP1R*, *PARK16*, *STX1B*, *FGF20*, *STBD1*, *GPNMB*, and *RIT2* [10], [17]–[18]. Hereafter, a large-scale replication and heterogeneity study also reported that *STK39* and *CCDC62/HIP1R* increased risk of PD in Asian and Caucasian populations [19]. In their study, Asian populations include a relatively large sample size from Hong Kong, Singapore, Taiwan, Korea, and Japan [19]. Notably, these studies did not include the Han Chinese population from mainland China.

To provide more evidence into genuine loci contributing to PD across diverse populations, we conducted a case-control study to examine the genetic associations of *STK39* (serine/threonine kinase 39, rs2102808) and *CCDC62/HIP1R* (Coiled-coil domain containing 62/Huntingtin-interacting protein 1-related, rs12817488) among Han Chinese in mainland China. We also performed further stratified analyses according to the age of onset and accomplished in-depth clinical characteristics analyses between the different genotypes for each locus.

## Materials and Methods

### Subjects

The study population included 1508 ethnic Han Chinese subjects comprising 783 sporadic PD patients (448 males, 335 females) and 725 controls (387 males, 338 females), all of whom were recruited from the Department of Neurology of the West China Hospital, Sichuan University. All patients (the mean age at onset 54.19±10.61, range 30–89) met United Kingdom Parkinson’s Disease Society Brain Bank clinical diagnostic criteria for PD as determined by two movement disorder specialists [20], except that patients who had an age at onset younger than 30 years. Sporadic PD was defined as PD without a family history of disease. Healthy control individuals (the mean age 55.26±12.85, range 30–91) of similar race, gender, and age from the same region as the PD patients were included. All subjects were divided into two subgroups according to the age of onset (<50 years of age and ≥50 years of age). The study was approved by the Ethics Committee of Sichuan University. All participants or their legal surrogates signed informed consent. DNA was extracted from venous blood using standard methods.

### Genetic Analysis

#### *STK39* (rs2102808)

Genotyping was performed using a matrix-assisted laser desorption/ionization time-of-flight (MALDI-TOF) mass spectrometry on a MassArray system (Sequenom, San Diego, CA, USA) for *STK39* rs2102808. Approximately 15 ng of genomic DNA was used to genotype each sample. Briefly, locus-specific polymerase chain reaction (PCR) and detection primers for the MALDI-TOF analysis of the *STK39* gene target fragment were designed using the MassArray Assay Design 3.0 software (Sequenom, San Diego, CA, USA). The sample DNAs were amplified by primers flanking the targeted sequence, followed by dephosphorylation and allele-specific primer extension. Eluted extension products were loaded to the dried matrix and finally subjected to MALDI-TOF mass spectrometry. The resultant data were analyzed by the Sequenom MassArray Typer software (Sequenom, San Diego, CA, USA).

#### *CCDC62/HIP1R* (rs12817488)

Genotyping was performed by using polymerase chain reaction followed by Restriction Fragment Length Polymorphism (PCR-RFLP) with MspI (Fementas) for *CCDC62/HIP1R* rs12817488. A 334 bps fragment was amplified by the following primer pair: 5′TTTGGAGGCTAAGGAAGGG3′ (forward) and 5′TTTGGGATGTGAAGTTTGGCA3′ (reverse). The PCR products were digested overnight with MspI at 37°C and electrophoresed on 2% agarose gel and visualized with ethidium bromide. The rs12817488 variant creates a MspI restriction site which cuts the normal 334 bps PCR products into fragments of 174 bps and171 bps. The AA genotype gave a single band of 334 bps, the AG genotype two bands of 334 bps+174 bps+171 bps and the GG genotype a mixed band of 174 bps+171 bps. 10% of samples were randomly selected for replication assays, the final results of which were completely concordant with the original results.

### Statistical Analysis

We assessed each locus for Hardy-Weinberg equilibrium (HWE) in cases and controls separately with an exact test. Differences between allelic and genotype frequencies in the patients and controls were compared through a Chi-squared test. The two-tailed Student’s t-test was used to compare the mean age between the different genotypes. A two-tailed *P*-value ≤0.05 was considered statistically significant. Statistical analysis was performed using the Statistical Package for the Social Sciences version 16.0 (SPSS, Chicago, IL, USA) for Windows. For power estimations we used NCSS-PASS software (NCSS, Kaysville, Utah, USA). Odd Ratios (ORs) of *STK39* rs2102808 and *CCDC62/HIP1R* rs12817488 were, respectively, 1.28 and 1.16 in the discovery phase of the original GWAS meta-analysis [10]. Additionally, based on the minor allele frequency (MAF) reported by our own study in Han Chinese controls, estimated power was high for *STK39* (∼91%), and modest for *CCDC62/HIP1R* (∼55%).

## Results

Data on a total of 1508 subjects including 783 cases and 725 controls were used for analysis. None of the single nucleotide polymorphisms (SNPs) departed from HWE in patients and controls. The allele frequencies of each SNP are summarized in Table 1 between cases and controls.

**Table 1:** Comparison of Allelic Frequencies between Cases and Controls.

Gene	SNP	CasesMAF	ControlsMAF	OR 95%(CI)	*P*-value
*STK39*	rs2102808	35.12%	33.86%	1.06 (0.91,1.23)	0.467
*CCDC62/* *HIP1R*	rs12817488	45.13%	48.45%	0.88 (0.76,1.01)	0.072

No significant differences were observed in the minor allele frequency among cases and controls at the two loci (rs2102808: OR = 1.06, 95% CI = 0.91, 1.23, *P* = 0.467; rs12817488: OR = 0.88, 95% CI = 0.76, 1.01, *P* = 0.072). In *STK39* (rs2102808), subjects with GT+TT genotypes was not significantly different from those with GG genotype (OR = 0.96, 95% CI = 0.78, 1.17, *P* = 0.657). At the same time, subjects with AG+GG genotypes have no significant differences compared to those with AA genotype at *CCDC62/HIP1R* rs12817488 (OR = 0.89, 95% CI = 0.71, 1.11, *P* = 0.286). In addition, further stratified analyses according to the age of onset also showed that the associations were not significant among the younger age group and the older age group in *STK39* and *CCDC62/HIP1R* (Table 2).

**Table 2:** Association between thetwo genetic loci and PD.

SNP	n	Gentype (%)		OR 95%(CI)	*P*-value
*STK39* rs2102808		GG	GT+TT		
PD^a^	783	335 (42.8)	448 (57.2)	0.96 (0.78,1.17)	0.657
EOPD^b^	262	104 (39.7)	158 (60.3)	0.96 (0.67,1.39)	0.851
LOPD^c^	521	231 (44.3)	290(55.7)	0.95 (0.74,1.21)	0.654
Controls	725	302 (41.7)	423 (58.3)		
Controls <50 yr	229	89 (38.9)	140 (61.1)		
Controls ≥50 yr	496	213 (42.9)	283(57.1)		
*CCDC62/* *HIP1R*rs12817488		AA	AG+GG		
PD	760	234 (30.8)	526 (69.2)	0.89 (0.71,1.11)	0.286
EOPD	260	78 (30.0)	182 (70.0)	0.78 (0.52,1.16)	0.220
LOPD	500	156(31.2)	344(68.8)	0.93(0.71,1.23)	0.622
Controls	708	200 (28.2)	508 (71.8)		
Controls <50 yr	224	56 (25.0)	168 (75.0)		
Controls ≥50 yr	484	144(29.8)	340(70.2)		

’s disease; LOPD, late onset Parkinson’s disease. EOPD, early onset Parkinson

^a^ Patients compared with controls by genotype.

^b^ EOPD Patients compared with controls (<50 years) by genotype.

≥50 years) by genotype.^c^ LOPD Patients compared with controls (

We also compared the clinical characteristics of PD cases that carried the minor allele with PD Cases who did not carry it (Table 3). However, there was no significant difference in the clinical presentation for gender, age of onset, onset symptoms at *STK39* rs2102808 and *CCDC62/HIP1R* rs12817488.

**Table:** Table3. Clinical Characteristics of PD Patients between minor allele carriers and non-carriers.

	Gentype		*P*-value
*STK39* rs2102808	GG	GT+TT	
General characteristics			
Gender			
Male (%)	190 (56.7)	258 (57.6)	0.807
Female (%)	145 (43.3)	218 (42.4)	
Age at onset^a^			
Total cohort	54.75±10.59	53.77±10.62	0.198
EOPD	42.47±4.82	42.44±4.88	0.955
LOPD	60.28±7.31	59.94±7.28	0.594
Onset symptoms			
Resting tremor (%)	167 (49.9)	230 (51.3)	0.589
Bradykinesia-rigidity (%)	126(37.6)	152 (33.9)	
Mixed symptoms (%)	16 (4.8)	21 (4.7)	
Others (%)^b^	26 (7.8)	45 (10.0)	
*CCDC62/HIP1R*rs12817488	AA	AG+GG	
General characteristics			
Gender			
Male (%)	128 (54.7)	308 (58.6)	0.321
Female (%)	106 (45.3)	218 (41.4)	
Age at onset^a^			
Total cohort	54.23±10.70	54.12±10.75	0.894
EOPD	42.23±5.10	42.53±4.78	0.648
LOPD	60.23±7.1	60.25±7.48	0.982
Onset symptoms			
Resting tremor (%)	112 (47.9)	270 (51.3)	0.313
Bradykinesia-rigidity (%)	92 (39.3)	180 (34.2)	
Mixed symptoms (%)	7 (3.0)	28 (5.3)	
Others(%)^b^	23 (9.8)	48 (9.1)	

± SD.^a^ Data are mean

^b^ Including pain, weakness, symptoms of autonomic dysfunction and so on.

## Discussion

The first large-scale meta-analysis of GWAS in PD identified 5 new PD genetic loci (*ACMSD*, *STK39*, *MCCC1/LAMP3*, *SYT11*, and *CCDC62/HIP1R*) [10]. Very recently, a case-control replication study provided support for all these loci in a relatively homogenous Scandinavian population [21]. Moreover, a large replication study [19] and acomprehensive meta-analysis from the PDGene database [22] also investigated the same associations for all 5 loci except *ACMSD* and subgroup analysis by ethnicity also showed similar effect size estimates for *STK39*, *CCDC62/HIP1R*, and *MCCC1/LAMP3* in Asian and Caucasian populations [19]. Besides, the other two intronic SNPs, rs3754775 and rs6740826 in *STK39*, showed the strongest evidence of association using an overall MAF threshold of 2% or higher to identify rare genetic variants in a relatively genetically homogeneous Ashkenazi Jewish population (OR = 2.12, 95% CI = 1.24, 3.62, *P* = 0.005) [8].

In view of the population-specific heterogeneity, we accomplished a case-control study included 1508 subjects to further explore the role of two newly identified genetic variants (*STK39* rs2102808 and *CCDC62/HIP1R* rs12817488) in risk of PD in a Han Chinese population from mainland China. Conversely, we are unable to replicate the association between *STK39* rs2102808 and *CCDC62/HIP1R* rs12817488 and PD susceptibility. Subgroup analyses by age of onset also showed no significant differences among different subgroups of the two loci. In addition, minor allele carriers cannot be distinguished from non-carriers based on their clinical features for gender, age of onset, onset symptoms at the two loci.

Our finding should be interpreted with caution as our modest sample size results in only modest power for some alleles with very small effect sizes. This is especially for *CCDC62/HIP1R* rs12817488. Future multi-site efforts and meta-analyses will be useful. Genetic heterogeneity across diverse populations may also explain the variances between studies. Our selected SNPs in our cohort might not be genuine functional variants, or the pattern of linkage disequilibrium (LD) might differ so that they are no longer “tagging” some unidentified functional variants effectively [21]. It is worth noting that the MAF for *STK39* rs2102808 differs markedly from that reported in the original meta-analysis of GWAS published in the Lancet in 2011 (0.35 vs. 0.13). This highlights possibility of population heterogeneity, which may underlie nonreplication in independent studies. Gene-environment and gene-geneinteractions may also be confounding variables? Taken together, sequencing or comprehensive tag-SNP genotyping and imputation across different populations will probably be utilized to disentangle how variation contributes to PD risk in future studies [21].

The *STK39* has been associated with hypertension, autism, and early-stage non-small-cell lung cancer [23]–[25]. The *STK39* encodes a serine/threonine kinase (SPAK/PASK/STE20-SPS1 homolog) of 547-amino acids with roles in stress signals, ion homoeostasis, and inflammatory status [26]–[29]. In fact, *CCDC62/HIP1R* involves two diverse genes [30]. *CCDC62* is linked to estrogen receptor transactivation, cyclin D1 expression in prostate cancer cells, and antibodies to this protein develop in a variety of cancers [31]–[32]. Additionally, the protein product of *HIP1R* is involved in clathrin-mediated endocytosis, actin dynamics, intrinsic cell death pathway, accurate distribution of chromosomes [33]–[36]. It appears to interacted with huntingtin to modulate polyglutamine-induced neuronal dysfunction in transgenic nematodes Huntington model [37]. However, the ture role of these genes awaits discovery in the pathophysiologic pathway of PD.

In conclusion, our study from mainland China demonstrates that *STK39* (rs2102808) and *CCDC62/HIP1R* (rs12817488) do not appear to influence PD risk. Subgroup analyses also showed no significant differences among different subgroups of the two loci. Furthermore, minor allele carriers cannot be distinguished from non-carriers based on their clinical features at the two loci. Additional replication studies in other populations and functional studies are warranted to better validate the role of the two new loci in PD susceptibility.
